# Egg quality, fatty acid composition and immunoglobulin Y content in eggs from laying hens fed full fat camelina or flax seed

**DOI:** 10.1186/s40104-016-0075-y

**Published:** 2016-03-03

**Authors:** Gita Cherian, Nathalie Quezada

**Affiliations:** Department of Animal and Rangeland Sciences, Oregon State University, Corvallis, OR 97331 USA

**Keywords:** Camelina seed, Egg quality, Flax seed, Immunoglobulin Y, n-3 fatty acids

## Abstract

**Background:**

The current study was conducted to evaluate egg quality and egg yolk fatty acids and immunoglobulin (IgY) content from laying hens fed full fat camelina or flax seed.

**Methods:**

A total of 75, 48-week-old Lohman brown hens were randomly allocated to 3 treatments, with 5 replicates containing 5 laying hens each replicate. The hens were fed corn-soybean basal diet (Control), or Control diet with 10 % of full fat camelina (Camelina) or flax seed (Flax) for a period of 16 wk. Hen production performance egg quality, egg yolk lipids, fatty acids and IgY were determined every 28 d during the experimental period.

**Results:**

Egg production was higher in hens fed Camelina and Flax than in Control hens (*P* < 0.05). Egg weight and albumen weight was lowest in eggs from hens fed Camelina (*P* < 0.05). Shell weight relative to egg weight (shell weight %), and shell thickness was lowest in eggs from hens fed Flax (*P* < 0.05). No difference was noted in Haugh unit, yolk:albumen ratio, and yolk weight. Significant increase in α-linolenic (18:3 n-3), docosapentaenoic (22:5 n-3) and docoshexaenoic (22:6 n-3) acids were observed in egg yolk from hens fed Camelina and Flax. Total n-3 fatty acids constituted 1.19 % in Control eggs compared to 3.12 and 3.09 % in Camelina and Flax eggs, respectively (*P* < 0.05). Eggs from hens fed Camelina and Flax had the higher IgY concentration than those hens fed Control diet when expressed on a mg/g of yolk basis (*P* < 0.05). Although the egg weight was significantly lower in Camelina-fed hens, the total egg content of IgY was highest in eggs from hens fed Camelina (*P* < 0.05).

**Conclusions:**

The egg n-3 fatty acid and IgY enhancing effect of dietary camelina seed warrants further attention into the potential of using camelina as a functional feed ingredient in poultry feeding.

## Background

Polyunsaturated fatty acids (PUFA) of the omega-3 (n-3) family have a wide range of demonstrated health-related benefits. These positive effects include: cardioprotective, anticancer, triglyceride and blood pressure lowering, immune health enhancing and their roles in growth and maturation of central nervous system [1–3]. Dietary n-3 fatty acids include α-linolenic acid (18:3 n-3), eicosapentaenoic acid (EPA, 20:5 n-3), docosapentaenoic acid (DPA, 22:5 n-3) and docosahexaenoic acid (22:6 n-3, DHA). α-linolenic acid is present in terrestrial oils (e.g. flax) while EPA, DPA, and DHA are present in marine oils. Due to the limited consumption of n-3 fatty acid-rich marine foods (e.g. fatty fish) in western countries, there is an increased interest in modifying animal food products with n-3 fatty acids [4, 5]. In this context, studies on enriching poultry foods (e.g. eggs, meat) with n-3 fatty acids through feeding strategies are a topic of continued interest [6, 7].

Oil seeds such as flax are usually incorporated into poultry diets due to their nutritional value such as metabolizable energy and crude protein content. Flaxseed contains about 34 % oil and high content of α-linolenic acid (>50 %) makes it a common feed ingredient for n-3 fatty acid enrichment [8, 9]. Due to the cost and limited availability of feed ingredients rich in n-3 fatty acids, alternate novel n-3 feedstuffs are explored. *Camelina sativa* or “false flax” or “wild flax” is an oilseed crop of the *Brassica* (Cruciferae) family that contains high levels of n-3 fatty acids [10, 11]. Although camelina has been cultivated since the Bronze Age, there is renewed interest in camelina as a feedstock for bio-fuel production. Investigations on the nutritional value in poultry feeding such as metabolizable energy [12, 13], digestibility [13, 14], egg and meat n-3 enriching [14–16] and antioxidant properties [17] of camelina coproducts (e.g. meal, cake) have been documented. However, no information is available on n-3 fatty acid enriching and immune-related effects of feeding full fat seeds of camelina in poultry. Considering the high demand of flax for human health-food uses, finding alternate sources of n-3 fatty acid-rich feeds will reduce production costs and will provide n-3 PUFA-enriched foods for human consumption. In this context, the objectives of the current study were to investigate the effect of feeding full fat camelina seeds to laying hens on egg quality, lipid and fatty acid composition, and egg production, during a 4 mo period of feeding trial. It was hypothesized that feeding camelina seeds will enhance n-3 fatty acid incorporation in eggs without affecting egg quality or hen production aspects. In addition, egg immunoglobulin Y (IgY) content was also determined, as our previous studies have shown that feeding linseed or fish oil rich in n-3 fatty acids led to significant increase in egg yolk IgY [18]. Chicken egg has been extensively studied as an important source of commercial antibodies and investigations on hen diet modulation to produce IgY may provide novel value-added nutraceuticals from eggs.

## Methods

An institutional animal care and use committee approved all experimental protocols to ensure adherence to Animal Care Guidelines.

### Birds, diets, and housing

The camelina and flax seed used in the current study were analyzed for gross energy, crude protein, amino acids, minerals and crude fat content at the University of Arkansas Poultry Science Central Analytical Laboratory and for fatty acids at Oregon State University (Table 1).

**Table 1:** Chemical composition and nutrient profile of full fat camelina and flaxseed

Nutrient composition	Camelina seed	Flax seed
Gross Energy, kcal/kg	6,090	6,530
Crude protein, %	25.8	19.0
Crude fat, %	38.9	42.0
Amino acid, %		
Aspartic acid	2.31	1.74
Threonine	0.96	0.63
Serine	0.88	0.66
Glutamic acid	4.26	3.32
Glycine	1.39	1.17
Alanine	1.16	0.85
Valine	1.46	0.81
Isolecucine	1.03	0.81
Leucine	1.72	1.10
Tyrosine	0.60	0.40
Phenylalanine	1.12	0.88
Lysine	1.25	0.77
Tryptophan	0.29	0.51
Histidine	0.52	0.39
Arginine	2.17	1.67
Cystine	0.67	0.39
Methionine	0.57	0.47
Fatty acid, %		
Palmitic (16:0)	6.46	5.81
Stearic (18:0)	2.49	3.47
Oleic acid (18:1)	17.54	15.61
Linoleic (18:2 n-6)	19.04	14.52
α-Linolenic (18:3 n-3)	33.21	60.08
Eicosenoic (20:1)	15.57	0.00
Total Saturated	9.04	9.08
Total Monounsaturated fatty acid	36.16	15.83
Total n-3 fatty acid	33.34	60.08
Total n-6 fatty acid	22.24	14.52
Minerals, ppm		
P	7,027	6,449
K	8,612	7,025
Ca	2,113	2,022
Mg	3,013	3,439
S	5,827	1,828
Na	28.8	168
Fe	99.2	61.3
Mn	20.2	41.7
Zn	45.9	53.4
Cu	6.73	8.97

A total of 75, 48-week-old brown laying hens (Lohman Brown) were randomly allocated to 3 treatment groups, with 5 replicates containing 5 laying hens each replicate. The birds were kept in individual cages (18 in × 21 in × 23 in) (width × length × height). The hens were fed corn-soybean basal diet (Control, negative control), or Control diet with 10 % of full fat camelina seeds (Camelina) or flax seed (Flax, positive control) (Table 2). All the diets were isocaloric and isonitrogenous. The hens originated from a study investigating the use of oil seeds in egg laying hens and were fed diets containing corn-soy-based control or camelina or flax meal for a period of four months [12]. Birds were taken off the experimental diets for 6 wk before the initiation of the current study. Water and feed were provided *ad libitum*. The laying hens did not receive any vaccines or drugs during the entire experimental period. The experimental diets were fed for a period of 16 wk. The birds were maintained on a 16 L:8D photoperiod and standard conditions of temperature and ventilation as per University Poultry Farm standard operating procedures.

**Table 2:** Experimental diet composition and calculated nutrient analysis

	Experimental diets^1^		
Ingredients, %	Control	Camelina	Flax
Corn grain	49.4	43.4	40.2
Soybean meal (49 % CP)	21.0	17.8	19.0
Wheat middling	19.8	19.0	21.0
Limestone	7.4	7.4	7.4
Dicalcium Phosphate	1.6	1.6	1.6
Premix^2^	0.4	0.4	0.4
Salt	0.4	0.4	0.4
Camelina seed	0.0	10.0	0.0
Flax seed	0.0	0.0	10.0
Nutrient composition			
Metabolizable energy, kcal/kg	2,800	2,800	2,800
Crude protein, %	18.0	18.0	18.0
Calcium, %	3.5	3.5	3.5
Available phosphorus, %	0.45	0.45	0.45
Lysine, %	0.89	0.89	0.80
Cystine, %	0.30	0.33	0.32
Methionine, %	0.27	0.38	0.37
Arginine, %	1.15	1.28	1.40
Threonine, %	0.63	0.64	0.63
Fatty acid composition, %			
Palmitic acid (16:0)	15.37	9.39	9.22
Plamitolieic acid (16:1)	1.10	0.52	0.08
Stearic acid (18:0)	3.35	2.59	3.50
Oleic acid (18:1)	19.91	18.70	17.52
Linoleic acid (18:2 n-6)	51.71	30.99	28.16
α-Linolenic acid (18:3 n-3)	5.19	22.84	39.76
Eicosaenoic acid (20:1)	0.66	11.55	0.54
Total saturated fatty acids	19.19	12.19	12.94
Total monounsaturated fatty acids	21.67	30.77	18.14
Total n-3 fatty acids	6.86	23.50	40.37
Total n-6 fatty acids	52.28	33.54	28.55
Totaln-6:n-3 fatty acids	7.69	1.43	0.71

^1^Control, Camelina, and Flax represent corn-soybean meal basal diet (Control); or basal diets containing *Camelina* seed at 10 % (Camelina) or flax seed at 10 % (Flax). ^2^Suppled per lb feed: Vit A, 740,000 IU; Vit D_3._ 440,000 ICU; Vit E 1,200 IU; Vit B_12_, 1.6 mg; Riboflavin, 800 mg; Pantothenic acid, 1,000 mg; Niacin, 6,000 mg; Menadione, 135 mg; Choline, 500 mg; Thiamine, 135 mg; Folic acid, 45 mg; Pyridoxine,180 mg; d-biotin, 0.15 mg; Ethoxyquin, 2.5 μg; Manganese, 2.5 %; Zinc, 92.4 mg; Selenium, 120 ppm; Zinc, 2.00 %; Choline, 50,000; Copper sulfate 2,000 ppm; Iodine 1.145 ppm; Iron 1.8 %

### Assessing Egg production and Egg quality

Egg production was recorded on individual hens and egg production (%) was calculated as total eggs divided by the total number of days and hens. A total of 10 eggs (2 from each replicate) from each treatment were taken every 28 day to assess egg quality parameters, fatty acid profile, lipid and IgY content. The eggs were weighed, and yolks were separated using an egg separator and were rolled on wet paper to remove any white albumen and were then weighed. Two yolks were pooled to obtain a sample size of 5 for fatty acid analyses. Albumen weight was calculated by subtracting yolk and shell weight from total egg weight. Haugh unit (HU) [19] (a measure of albumen thickness) was determined by measurement albumen height by using a tripod micrometer. The Haugh units (HU) were calculated by the formula HU = 100 log (H + 7.57 − 1.7 W0.37), where H is the average albumen height (mm) and W is the weight of the egg (g). Shell was wiped clean and weighed. Shell thickness was measured using an electronic micrometer. Yolk color was measured by comparing yolk color to the Roche yolk color fan.

### Total lipid and fatty acid analysis

About 2 g of feed or egg yolk, was taken for total lipid extraction using chloroform: methanol (2:1) following the method of Folch et al. [20]. Fatty acid methyl esters were prepared from total lipid extract using methanolic HCl [21]. An internal standard (23:0) (Matreya, PA) was used for fatty acid quantification. The analysis of fatty acids were performed with an Agilent 6890 gas chromatograph (Agilent Technologies, CA) equipped with an autosampler, flame-ionization detector, and fused-silica capillary column, 30 m × 0.25 mm × 0.2 μm film thickness (Supelco, PA). Each sample (1 μL) was injected with helium as a carrier gas onto the column programmed for increased oven temperatures (the initial temperature of 110 °C was held for 0.5 min, then increased by 20 °C/min to 190 °C, held for 7 min, and then increased at 5 °C/min to 210 °C and held for 8 min). Inlet and detector temperatures were both 250 °C. Peak areas and fatty acid percentages were calculated using Agilent ChemStation software (Agilent Technologies, CA). Fatty acid methyl esters were identified by comparison with retention times of authentic standards (Matreya, PA) and were expressed as percentages of total fatty acid methyl esters or as mg/egg.

### Determination of IgY concentration in Egg yolk

Egg immunoglobulin Y was isolated from egg yolk by the method described earlier [22]. Briefly, about 150 to 200 mg of pooled egg yolk was diluted 1:6 (v/v) with acidified deionized water (pH 2.5), vortexed well, and stored at 4 °C. After overnight refrigeration, samples were centrifuged at 10,062 × *g* at 4 °C for 15 min and the supernatants were collected and egg IgY contents were quantified by ELISA using rabbit anti-chicken IgG (Rockland Inc., Gilbertsville, PA) as described earlier [23]. Egg weight and yolk weight were used to calculate IgY in milligrams per gram of egg yolk or total IgY per egg.

### Statistical analyses

The effects of diet on hen production performances, egg quality, egg total lipids, fatty acid composition and IgY content were analyzed by two-way ANOVA using SAS 9.4 [24]. Diet and week were the main factors. Each cage was considered as an experimental unit. Significant differences among treatment means were analyzed by Tukey’s HSD test at *P* < 0.05 [25].

## Results and discussion

The nutrient profiles of the two oil seeds (camelina and flax) are reported in Table 1. The gross energy content of camelina and flax seed were 6,090 and 6,530 kcal/kg. The crude protein content was higher in camelina seed (25.8 %) than flax seed (19.0 %). The crude fat constituted 38.9 % in camelina seed compared to 42.0 % in flax seed. The protein of camelina and flax seed contained several essential amino acids such as threonine, methionine, valine, isoleucine, leucine, lysine, and phenylalanine (Table 1). The α-linolenic acid (18:3 n-3) in camelina seed was comparatively lower than that of flax seed (Table 1). Linoleic acid (18:2 n-6) was higher in camelina seed than flax seed. Camelina seeds also had very high levels of eicosaenoic acid (20:1). Total saturated fatty acids (14:0 + 16:0 + 18:0) constituted 9.04 and 9.08 % for camelina and flax seed, respectively. Palmitic acid (16:0) was major saturated fatty acid followed by stearic acid (18:0) in both oil seeds. The high protein, energy and n-3 and n-6 fatty acid content of camelina seed makes it a potentially suitable source of plant protein and essential n-6 and n-3 fatty acid source in poultry diets. Among the several minerals in camelina seed, potassium was the major mineral followed by phosphorus, sulfur, magnesium and calcium.

The experimental diet composition, nutrient composition and fatty acid profile of the diets are shown in Table 2. Inclusion of camelina and flax seed led to increase in α-linolenic acid in the diet along with a reduction in saturated fatty acids. With a greater proportion of n-3 PUFAs, the proportion of n-6 PUFA decreased in the diets. Thus, the ratio of n-6 PUFAs to n-3 PUFAs averaged 7.69, 1.43 and 0.71 for Control, Camelina and Flax, respectively. Oleic acid in the diets constituted 19.9, 18.7 and 17.5 for Control, Camelina and Flax, respectively. Total monounsaturated fatty acids (16:1 + 18:1 + 20:1) were higher in Camelina than Flax and Control due to the presence of eicosaenoic acid.

### Hen production and Egg quality

A 4 % increase in egg production was noted in hens fed the Camelina and Flax (*P* < 0.05) (Table 3). No difference in egg production could be observed between hens fed Camelina and Flax. A significant increase feed consumption (g/d) was observed in hens fed Camelina and Flax compared to Control hens (*P* < 0.05). Egg weight and albumen weight was lowest in eggs from hens fed Camelina (*P* < 0.05). Shell weight relative to egg weight (shell weight %), and shell thickness was lowest in eggs from hens fed Flax (*P* < 0.05). No difference was noted in Haugh unit, yolk:albumen ratio, and yolk weight. Reduction in egg and albumen weight observed in the current study corroborated with our previous studies on feeding camelina meal in laying hen diets [12]. Camelina belongs to the *Brassica* family and species of this family are high in non-starch polysaccharides, glucosinolates and other antinutritional factors such as trypsin inhibitor [26]. While assessing the digestibility in hens fed diets containing camelina meal we observed a significant reduction in crude protein digestibility [12]. It is not known if the protein digestibility was impaired in the current study affecting nitrogen retention and egg albumen weight. Most studies on camelina in layer hen feeding were on coproducts such as meal or cake [14, 26, 27]. To author’s knowledge, no studies have shown impact of dietary full fat camelina seeds on egg characteristics. Inclusion of flax seeds in laying hens diets significantly decreased the yolk color (Table 3). The yellow color of egg yolk is produced by xanthophyll pigments, derived from diet. In this study, we observed lighter egg yolk color from laying hens fed flax seeds, probably due to lesser pigments in the diets. Inclusion of flax seed led to reduction (~9 %) in the amount of corn compared to Control diets used in the diets consequently affecting the pigment content and leading to paler yolk. However, we did not measure the pigment content in the experimental diets.

**Table 3:** Effect of dietary inclusion of camelina and flax seeds on production performance of brown laying hens during 16 wk of feeding period

Egg parameters	Dietary treatments^1^			Pooled SEM	*P* value		
	Control	Camelina	Flax		Diet	Week	Diet x Week
Egg production, %	87.67^b^	92.81^a^	92.65^a^	0.03	0.012	0.408	0.307
Feed consumption, g/d	118.60^b^	128.04^a^	130.64^a^	2.60	0.001	0.260	0.937
Egg weight, g	63.43^a^	60.43^b^	63.65^a^	0.87	0.017	0.006	0.890
Yolk weight, g	15.99	15.32	15.62	0.59	0.22	0.356	0.664
Shell weight, g	6.95	6.76	6.64	0.11	0.148	0.993	0.481
Albumen weight, g	40.49^a^	38.35^b^	41.40^a^	0.67	0.006	0.002	0.942
Shell thickness, mm	44.08^a^	42.73^ab^	41.00^b^	0.64	0.005	0.004	0.035
Yolk weight, %	25.22	25.36	24.55	0.33	0.182	0.243	0.665
Yolk color	5.67^a^	5.40^ab^	5.17^b^	0.11	0.009	0.001	0.001
Shell weight, %	10.96^a^	11.21^a^	10.49^b^	0.15	0.005	0.006	0.634
Albumen weight, %	63.81^b^	63.42^b^	64.96^a^	0.37	0.012	0.021	0.821
Haugh unit	63.11	68.68	66.12	2.20	0.208	0.122	0.545
Yolk:albumen	0.40	0.40	0.38	0.01	0.106	0.165	0.704

^a,b^Means within a row with no common superscript differ significantly (*P* < 0.05). Pooled SEM = standard error of mean

^1^Control, Camelina and Flax represent corn-soybean meal basal diet (Control); or basal diets containing camelina (Camelina) or flax seeds (Flax) at 10 %

### Total lipids and fatty acid composition of Egg yolk

Total lipid content and fatty acids profiles of egg yolk of laying hens fed Camelina and Flax is presented in Table 4. No difference was observed in the total fat content of eggs and was 33.04, 33.30, and 32.86 % for Control, Camelina and Flax, respectively (*P* > 0.05). As the hens, aged a significant increase in egg lipids was noted. A significant diet × week interaction was observed for egg lipids. The alteration of egg lipids through diet has long been studied but still receives a lot of attention because of its implications for human health [28, 29]. Furthermore, consumers are becoming increasingly interested in purchasing eggs from hens fed plant-based feeds containing high amount of n-3 polyunsaturated fatty acids.

**Table 4:** Effect of camelina and flax seed in the diet of layer birds on egg fatty acid composition

	Dietary Treatments^d^				*P*-Value		
Egg lipids, %	Control	Camelina	Flax	Pooled SEM	Diet	Month	Diet x Week
Palmitic acid (16:0)	25.42	25.80	24.86	0.30	0.09	0.33	0.59
Palmitoleic acid (16:1)	5.85	5.99	5.48	0.14	0.25	0.006	0.97
Stearic acid (18:0)	7.71	7.78	7.63	0.15	0.80	0.65	0.28
Oleic acid (18:1)	47.92^a^	45.52^b^	46.45^b^	0.47	0.004	0.25	0.90
Linoleic acid (18:2 n-6)	9.27	9.38	10.35	0.54	0.31	0.080	0.33
α-Linolenic acid (18:3 n-3)	0.42^b^	1.53^a^	1.49^a^	0.11	0.001	0.001	0.02
Eicosenoic acid (20:1)	0.31^b^	0.47^a^	0.28^b^	0.02	0.001	0.071	0.20
Arachidonic acid (20:4 n-6)	1.69^a^	1.31^b^	1.15^b^	0.08	0.0003	0.022	0.07
Docosapentaenoic (22:5 n-3)	0.06^c^	0.27^a^	0.19^b^	0.02	0.0001	0.009	0.81
Docosahexaenoic acid (22:6 n-3)	0.66^b^	1.25^a^	1.35^b^	0.05	0.0001	0.46	0.08
Total saturated fatty acids	33.62	34.06	32.96	0.33	0.085	0.465	0.39
Total monounsaturated fatty acids	54.08^a^	51.99^b^	52.22^b^	0.45	0.004	0.156	0.81
Total omega-6 fatty acids	11.10	10.82	11.72	0.53	0.482	0.689	0.24
Total omega-3 fatty acids	1.19^b^	3.12^a^	3.09^a^	0.17	0.001	0.005	0.03
Total omega-6:omega-3	5.99^a^	2.45^b^	2.80^b^	0.29	0.001	0.001	0.001
Total lipids, %	33.04	33.30	32.86	0.41	0.75	0.001	0.03

^a,b,c^Means within a row with no common superscripts differ significantly (*P* < 0.05). Pooled SEM = pooled standard error of mean. ^d^Control, Camelina and Flax represent corn-soybean meal basal diet (Control); or basal diets containing camelina (Camelina) or flax seeds (Flax) at 10 %

Total saturated fatty acids (14:0 + 16:0 + 18:0 + 20.0); Total monounsaturated fatty acids (16:1 + 18:1 + 20:1 + 22:1); Total n-6 polyunsaturated fatty acids (18:2 n-6 + 20:4 n-6 + 20:3 n-6 + 22:4 n-6 + 22:5 n-6); Total n-3 polyunsaturated fatty acids (18:3 n-3 + 22:5 n-3 + 22:6 n-3)

**Fig. 1:**
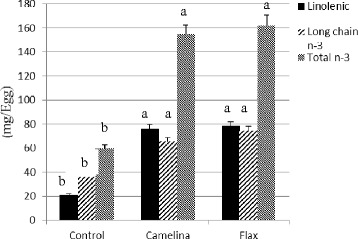
α-Linolenic acid, long chain n-3 fatty acid and total n-3 fatty acid supplied through eggs from layer hens fed Control, Camelina or Flax. ^a-b^Means between diets and within a fatty acid type without a common letter differ significantly (*P* < 0.05). Control, Camelina and Flax represent corn-soybean meal basal diet (Control); or basal diets containing camelina (Camelina) or flax seeds (Flax) at 10 %

In the current study it was observed that feeding oil seeds such as camelina and flax led to significant changes in egg yolk fatty acid profile (Table 4, Fig. 1). Palmitic acid was the major saturated fatty acid in eggs followed by stearic acid. No effect of dietary treatments on egg palmitic acid (*P* = 0.09) and total saturated fatty acids (14:0 + 16:0 + 18:0 + 20:0) was observed (*P* < 0.085). Oleic acid was lower in eggs from hens fed Camelina and Flax when compared to Control (*P* < 0.05). Eicosenoic (20:1) was higher in egg yolks from hens fed Camelina than Flax and Control which is not surprising because the camelina seeds contained 15.7 % of eicosaenoic acid. Overall, addition of oil seeds in the hen diet led to reduction in total monounsaturated fatty acids (16:1 + 18:1 + 20:1) in egg. The inclusion of full fat oil seeds increased the concentration of α-linolenic acid, DPA and DHA in the egg yolk (*P* < 0.05). The increase in DPA and DHA in eggs from hens fed Camelina and Flax suggest that laying hens can desaturate and elongate α-linolenic acid, the parent n-3 fatty acid precursor to form long chain 22-C n-3 fatty acids. The highest incorporation of DPA was observed in eggs from hens fed Camelina. Docosapentaenoic acid is usually the major long chain n-3 fatty acid in poultry tissues fed plant-based sources with high α-linolenic acid and the current study is in agreement with other reported research [30]. The n-3 fatty acid content of eggs from Camelina in the current study was lower than our previous reported research on feeding camelina meal to hens. Full fat seeds of the *Brassicca* family like camelina are high in cell wall non starch polysaccharides which limit exposure of seed oils to hen digestive enzymes affecting digestibility and egg incorporation.

No difference was observed in the linoleic acid and total n-6 fatty acids content of eggs, although the oil seed diets had lower linoleic acid than Control. However, arachidonic acid and total n-6:n-3 fatty acid ratio was lowest in eggs from Camelina and Flax (*P* < 0.05). The findings of the present study are in agreement with our previous reported research [14, 21] showing an increase in α-linolenic acid, followed by decreased in arachidonic acid and n-6:n-3 ratio in eggs from hens fed flax seeds or camelina meal. The high linolenic acid and lower ratio of n-6:n-3 fatty acids in Camelina and Flax may have decreased the competition of linoleic with α-linolenic acid for deasturase and elongase enzymes involved in bioconversion to linoleic to arachidonic acid resulting in reduced egg content of arachidonic acid.

Consumer interest in food products enriched with functional nutrients such as n-3 fatty acids are growing rapidly and egg is a suitable vehicle for delivering health-promoting omega-3 fatty acids [31]. For example, consumption of two eggs (1 serving) from hens fed Camelina and Flax could provide over 300 mg of total n-3 fatty acids with over 150 mg of it being long chain 22-C n-3 fatty acids (Fig. 1). The average per capita intake of long chain n-3 PUFA is approximately 0.1–0.2 g per day in North America [32]. The Dietary Guidelines for Americans recommends 0.65 g and the WHO recommends daily intake of 300–500 mg of EPA and DHA [32]. Therefore, consuming two eggs from hens fed Camelina could provide over 30 to 50 % of the extra needed long chain n-3 PUFA in the diet. The role of dietary flax seeds in enriching egg n-3 fatty acids is well documented [9, 21]. However, to the author’s knowledge, the effect of feeding full fat camelina seeds on egg yolk fatty acid composition is not known.

### Egg immunoglobulin Y

**Fig. 2:**
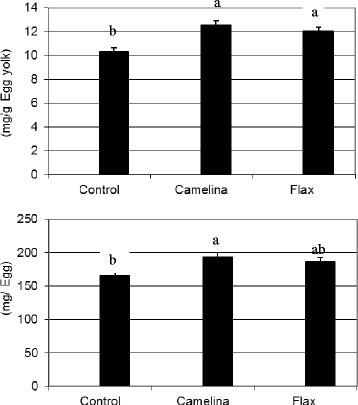
Effect of camelina and flax seed in the diet of layer birds on egg immunoglobulinY (IgY) content. ^a-b^Means between diets without a common letter differ significantly (*P* < 0.05). Control, Camelina and Flax represent corn-soybean meal basal diet (Control); or basal diets containing camelina (Camelina) or flax seeds (Flax) at 10 %

ImmunoglobulinY concentration of egg yolk and total IgY in the egg is shown in Fig. 2. The hens fed Camelina and Flax had the higher IgY concentration than those hens fed Control diet when expressed on a mg/g of yolk basis (*P* < 0.05). Although the egg weight was significantly lower in Camelina-fed hens, the total egg content of IgY was highest in eggs from hens fed Camelina (*P* < 0.05). Previously we reported significant increase in hen serum IgG and egg IgY in hens fed diets high in n-3 fatty acids such as linseed or fish oil when compared to sunflower oil or animal tallow [18]. The ratio of dietary n-6 to n-3 PUFA is decisive factor in modulation of IgG formation by immune cells and subsequently transportation in the plasma and deposition to egg yolk. In the current study dietary n-6 to n-3 fatty acid ratio was over 5 to 11-fold higher in Control compared to Camelina and Flax. Changes in dietary n-6 to n-3 fatty acid ratios can induce significant alterations in immune cell PUFA composition and eicosanoid production in layer hens [33]. Our previously reported research has shown that high dietary n-3 fatty acids can lead to decreased production of arachidonic acid-derived eicosanoids such as prostaglandin E2 as well as interleukins (IL-6), which can affect the production of immunoglobulins [34, 35]. No information on feeding full fat camelina seeds on egg yolk IgY is known. Over the last decade, use of chicken eggs for harvesting antibodies for therapeutic use has been attempted. In this context, use of diet in modulating egg IgY may open new avenues for isolating value-added functional ingredients for pharmaceutical or other nutraceutical purposes.

## Conclusions

In conclusion, the results of the present study indicate that feeding camelina or flax seeds enhanced egg production while enriching eggs with health-promoting n-3 fatty acids. The effect that feeding camelina increasing IgY in egg warrants further attention into the immunomodualting properties of this oil seed. Camelina is a nonfood crop and considering the lack of competition of camelina for human food uses, its potential to develop as a functional livestock feed for food enrichment as well as animal health deserves further research.
